# SIK2 regulates fasting-induced PPARα activity and ketogenesis through p300

**DOI:** 10.1038/srep23317

**Published:** 2016-03-17

**Authors:** Zhen-Ning Zhang, Lulu Gong, Sihan Lv, Jian Li, Xiaolu Tai, Wenqi Cao, Bing Peng, Shen Qu, Weida Li, Chao Zhang, Bing Luan

**Affiliations:** 1 Department of Endocrinology, Shanghai Tenth People’s Hospital; Translational Medical Center for Stem Cell Therapy & Institute for Regenerative Medicine, Shanghai East Hospital, Tongji University, Shanghai, China; 2 Translational Medical Center for Stem Cell Therapy & Institute for Regenerative Medicine, Shanghai East Hospital, School of Life Sciences and Technology, Tongji University, Shanghai, China; 3 Department of Endocrinology, Shanghai Tenth People’s Hospital, School of Medicine, Tongji University, Shanghai, China

## Abstract

Fatty acid oxidation and subsequent ketogenesis is one of the major mechanisms to maintain hepatic lipid homeostasis under fasting conditions. Fasting hormone glucagon has been shown to stimulate ketone body production through activation of PPARα; however, the signal pathway linking glucagon to PPARα is largely undiscovered. Here we report that a SIK2-p300-PPARα cascade mediates glucagon’s effect on ketogenesis. p300 interacts with PPARα through a conserved LXXLL motif and enhances its transcriptional activity. SIK2 disrupts p300-PPARα interaction by direct phosphorylation of p300 at Ser89, which in turn decreases PPARα-mediated ketogenic gene expression. Moreover, SIK2 phosphorylation defective p300 (p300 S89A) shows increased interaction with PPARα and abolishes suppression of SIK2 on PPARα-mediated ketogenic gene expression in liver. Taken together, our results unveil the signal pathway that mediates fasting induced ketogenesis to maintain hepatic lipid homeostasis.

Under fasting state, increased glucagon concentration induces gluconeogenesis for glucose production and hepatic fatty acid oxidation and ketogenesis for ketone body (acetoacetate and β-hydroxybutyrate) production^1^. Glucose and ketone bodies provide most of the energy needs of the brain during fasting and starvation^2^.

Glucagon-induced gluconeogenesis program has been well established. Gluconeogenesis is controlled by key transcription factors, CREB and FOXO1, at transcriptional level. By binding to its receptor, glucagon triggers cAMP production through adenylate cyclase. cAMP, as a second messenger, promotes protein kinase A (PKA) activation, which in turn phosphorylates CREB and promotes its transcriptional activity, CBP/p300 co-activator binding and expression of gluconeogenic genes, such as *Pepck* and *G6Pase*^3^. Besides, PKA could phosphorylate and inactivate salt-inducible kinases (SIKs; SIK1, 2, 3), which phosphorylate and suppress CREB-regulated transcription coactivator (CRTC)^4^ and CBP/p300^5^. Recent studies have also demonstrated that SIKs could phosphorylate and suppress class II HDACs, which deacetylate and inactivate FOXO1^6^,^7^. SIKs, AMPK superfamily members, thus play an important role in the regulation of gluconeogenesis.

Hepatic fatty acid oxidation and subsequent ketogenesis is mainly controlled by peroxisome proliferator–activated receptor α (PPARα) at transcriptional level^8^,^9^,^10^. PPARα is expressed predominantly in liver and is activated by glucagon^11^,^12^. PPARα binds to DNA-response elements as a heterodimer with the retinoid X receptor (RXR) to regulate the transcription of key genes involved in fatty acid oxidation and ketogenesis, such as *acyl-CoA oxidase* (*Aox*) and *hydroxymethylglutaryl-CoA synthase 2* (*Hmgcs2*)^13^,^14^. PPARα knockout mice exhibit decreased levels of fatty acid oxidation and ketogenesis during fasting and starvation^9^. Despite the critical role of the PPARα in the control of ketogenesis, the upstream pathway regulating this transcription factor still remains unknown.

In this report, we show that SIK2 could also regulate PPARα activity and ketogenesis through p300. p300 interacts with PPARα and functions as co-activator of PPARα. SIK2 directly phosphorylated p300 on Ser89, which in turn decreased the binding of p300 with PPARα. Thus, loss of SIK2 activity increased PPARα activity and stimulated ketone body production. While overexpression of SIK2 eliminated PPARα activities, expression of p300 phosphorylation defective mutant rescued the SIK2 phenotype. Overall, our results identified the SIKs-p300-PPARα cascade as the upstream signal pathway that mediated fasting induced activation of PPARα. SIK2-dependent regulation of p300 function could be important for the modulation of fatty acid oxidation and ketogenesis in obesity and insulin-resistance states.

## Results

### Fasting signal induces PPARα activation and ketogenesis

**Figure 1:**
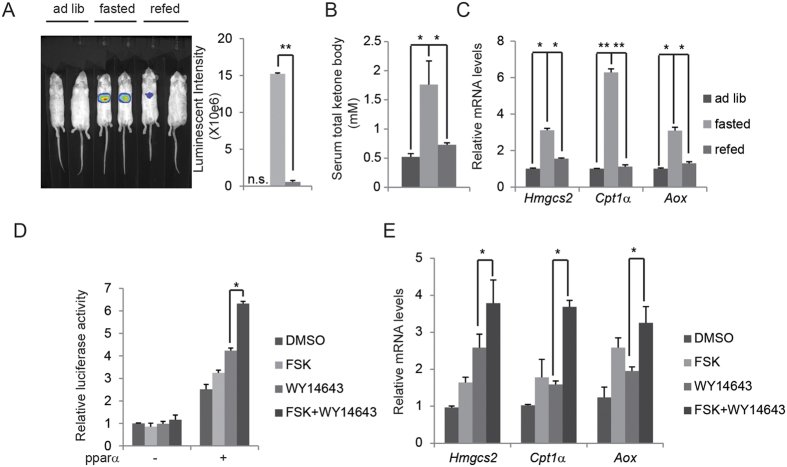
Fasting signal induces PPARα activation and ketogenesis. (**A–C**) Effect of fasting on hepatic *Aox*-PPRE-luciferase reporter activity (**A**), serum total ketone body (**B**) as well as mRNA amounts for hepatic ketogenic genes (**C**) in mouse liver. Quantification of luminescent intensity of (**A**) was shown. (**D**) *Aox*-PPRE-luciferase reporter activity in primary hepatocytes infected with Ad-PPARα or not. Effect of FSK, WY14643 or both was shown. E, Effect of FSK, WY14643 or both on mRNA amounts of ketogenic genes in HepG2 cells.

First, we tested whether PPARα activity was induced by fasting signal *in vivo*. *Aox* is a typical PPARα target gene mediating fatty acid oxidation and ketogenesis and its promoter contains PPARα binding motif (PPRE)^15^. We monitored PPARα activity in liver by *in vivo* imaging with adenovirus-containing *Aox* promoter with PPRE-luciferase reporter (Ad-*Aox*-PPRE-luc). Fasting increased hepatic Ad-*Aox*-PPRE-luc activity 15-fold over that of refed mice (Fig. 1A). Circulating ketone body concentration as well as PPARα target gene expression including *Hmgcs2*, *Cpt1α* and *Aox*, was correspondingly elevated in livers of fasted mice, whereas refeeding suppressed ketone production and gene expression of *Hmgcs2*, *Cpt1α* and *Aox* (Fig. 1B,C). During fasting stage, glucagon stimulates cAMP production in hepatocytes, prompting us to test the role of this second messenger in mediating PPARα activity. As shown in Fig. 1D, while PPARα agonist WY14643 increased Ad-*Aox*-PPRE-luc activity in primary hepatocytes, co-treatment with cAMP stimulus Forskolin (FSK) further promoted its activity (Fig. 1D). Consistently, the stimulation of PPARα target gene expression by PPARα activator WY14643 was also potentiated by FSK co-treatment (Fig. 1E). These results indicate that cAMP signaling under fasting state promotes hepatic PPARα activity both *in vivo* and *in vitro*.

### SIK2 inhibits PPARα activation and ketogenesis

**Figure 2:**
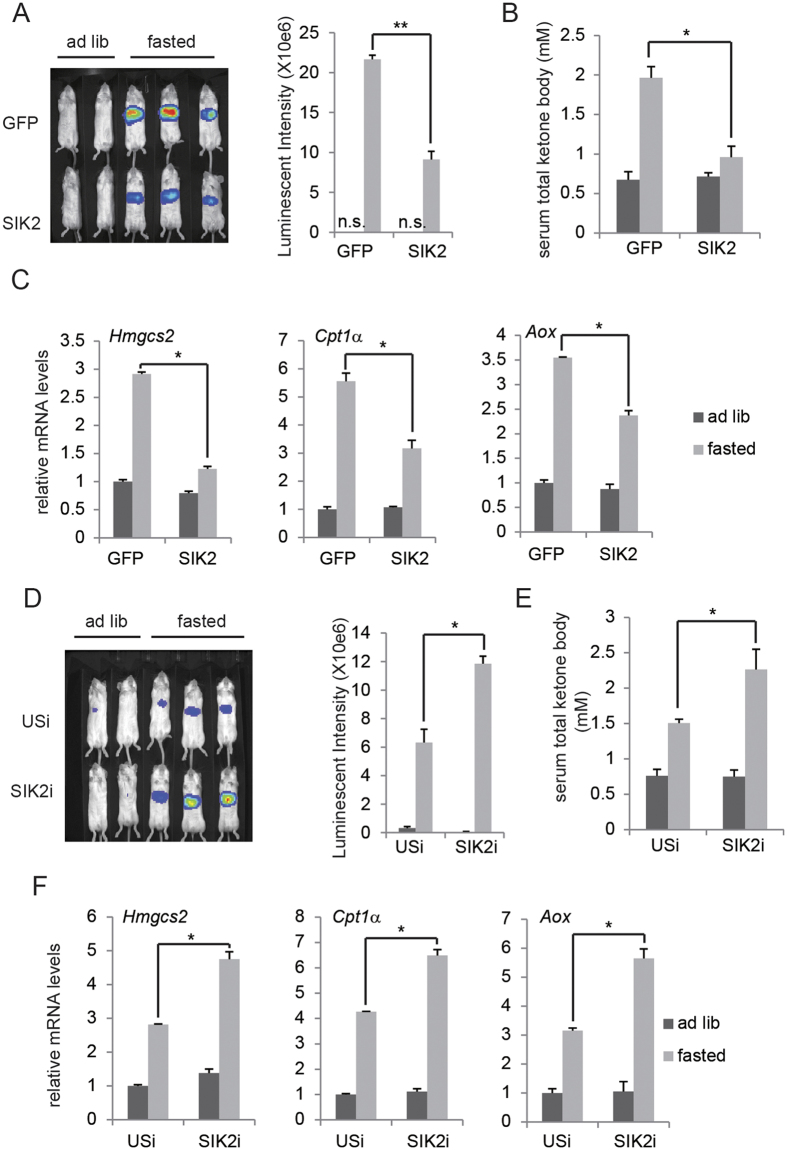
SIK2 inhibits PPARα activation and ketogenesis in liver. (**A–C**) Effect of fasting on hepatic *Aox*-PPRE-luciferase reporter activity (**A**), serum total ketone body (**B**) as well as mRNA amounts for hepatic ketogenic genes (**C**) in mice injected with Ad-GFP or Ad-SIK2. Quantification of luminescent intensity of (**A**) was shown. (**D–F**) Effect of fasting on hepatic *Aox*-PPRE-luciferase reporter activity (**D**), serum total ketone body (**E**) as well as mRNA amounts for hepatic ketogenic genes (**F**) in mice injected with Ad-USi or Ad-SIK2i. Quantification of luminescent intensity of (**D**) was shown.

On the basis of the role in regulating glucagon-induced hepatic gluconeogenesis^4^,^16^, the salt inducible kinases (SIKs) would be expected to modulate PPARα activity and ketogenesis with similar mechanism. To test this notion, we first assessed effect of SIKs on *Fgf21*-PPRE-luc activity. Of the three family members (SIK1, SIK2 and SIK3), SIK2 is the most highly expressed in liver (Fig. S1A). When expressed in HepG2 cells, all three members of SIK family were able to inhibit *Fgf21*-PPRE-luc activity upon exposure to WY14643 plus FSK and SIK2 showed most potent effect (Fig. S1B and S1C). Moreover, dominant-positive SIK2 (SIK2 S587A)^17^ exhibited more dramatic inhibition on *Fgf21*-PPRE-luc activity compared to WT SIK2, while kinase-defective SIK2 (SIK2 K49M)^18^ showed no effect at all (Fig. S1D and S1E). On the contrary, when reduction of endogenous SIK2 expression through SIK2 RNAi, WY14643 plus FSK-induced *Fgf21*-PPRE-luc activity was increased (Fig. S1F and S1G). As a result, SIK2 expression in HepG2 cells greatly inhibited WY14643 plus FSK-induced ketogenic gene expression (Fig. S1H), while RNAi mediated SIK2 knockdown promoted ketogenic gene expression (Fig. S1I). We tested the importance of SIK2 in modulating hepatic PPARα activity and ketogenesis *in vivo* by using adenovirus-mediated SIK2 expression (Ad-SIK2) and RNAi knockdown (Ad-SIK2i). Ad-SIK2 expression reduced hepatic *Aox*-PPRE-luc activity (Fig. 2A) as well as circulating ketone concentration (Fig. 2B) and PPARα target gene expression (Fig. 2C) under fasting state comparing to Ad-GFP expression. On the contrary, when SIK2 expression was knocked down by Ad-SIK2i, hepatic *Aox*-PPRE-luc activity (Fig. 2D) as well as circulating ketone concentration (Fig. 2E) and PPARα target gene expression (Fig. 2F) was promoted under fasting state comparing to Ad-USi injected mice, revealing the importance of SIK2 in this setting.

### p300 promotes PPARα activation and ketogenesis through interaction

**Figure 3:**
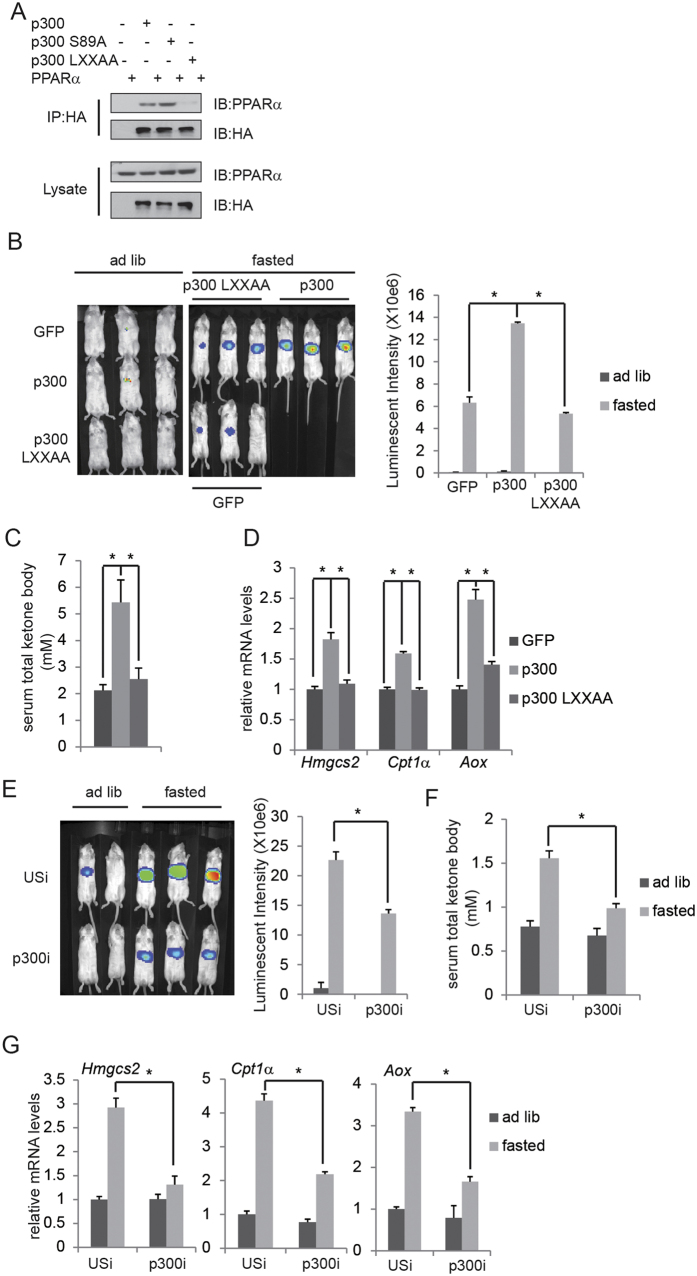
p300 interacts with PPARα and promotes its activation and ketogenesis. (**A**) Immunoblot showing recovery of PPARα from IPs of p300 prepared from HepG2 transfected with HA-tagged p300, HA-tagged p300 S89A, HA-tagged p300 LXXAA and PPARα expression vectors as indicated. (**B–D**) Effect of fasting on hepatic *Aox*-PPRE-luciferase reporter activity (**B**), serum total ketone body (**C**) as well as mRNA amounts for hepatic ketogenic genes (**D**) in mice injected with Ad-GFP or Ad-p300. Quantification of luminescent intensity of (**B**) was shown. (**E–G**) Effect of fasting on hepatic *Aox*-PPRE-luciferase reporter activity (**E**), serum total ketone body (**F**) as well as mRNA amounts for hepatic ketogenic genes (**G**) in mice injected with Ad-USi or Ad-p300i. Quantification of luminescent intensity of (**E**) was shown.

Co-activator p300 has been shown to interact with PPARα directly through its LXXLL domain to promote PPARα activity^19^. We were able to confirm the binding of PPARα with p300 but not with the LXXAA mutant of p300 in HepG2 cells (Fig. 3A). When expressed in mouse liver through adenovirus, Ad-p300 but not Ad-p300 LXXAA mutant promoted hepatic *Aox*-PPRE-luc activity (Fig. 3B) as well as circulating ketone concentration (Fig. 3C) and PPARα target gene expression (Fig. 3D). On the contrary, p300 knockdown by Ad-p300i attenuated hepatic *Aox*-PPRE-luc activity (Fig. 3E) as well as circulating ketone concentration (Fig. 3F) and PPARα target gene expression (Fig. 3G) comparing with Ad-USi injected mice. Consistent with these *in vivo* data, p300 expression and knockdown regulated WY14643 plus FSK-induced *Fgf21*-PPRE-luc activity and PPARα target gene expression in HepG2 cells respectively (Fig. S2).

### SIK2 inhibits PPARα activation through p300

**Figure 4:**
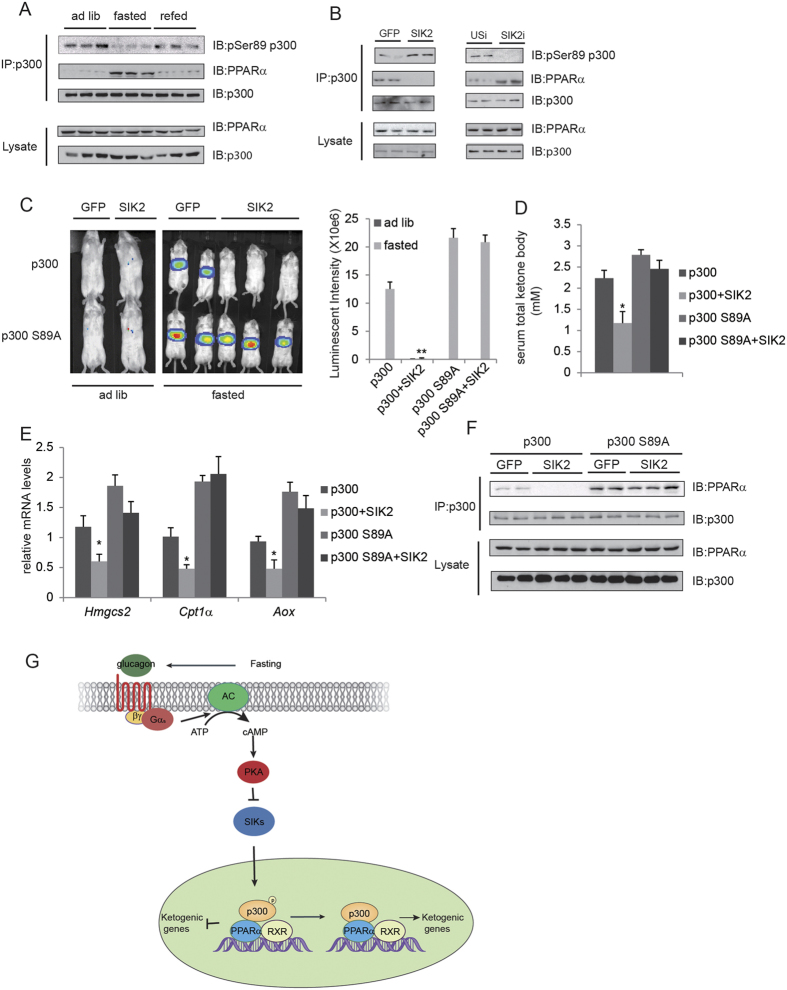
SIK2 regulates PPARα activation and ketogenesis through p300 phosphorylation. (**A**) Immunoblot showing p300 Ser89 phosphorylation and recovery of PPARα from IPs of p300 prepared from liver under ad lib, fasted or refed conditions as indicated. (**B**) Immunoblot showing p300 Ser89 phosphorylation and recovery of PPARα from IPs of p300 prepared from liver infected with Ad-GFP, Ad-SIK2 or Ad-USi, Ad-SIK2i as indicated. (**C–E**) Effect of fasting on hepatic *Aox*-PPRE-luciferase reporter activity (**C**), serum total ketone body (**D**) as well as mRNA amounts for hepatic ketogenic genes (**E**) in mice injected with Ad-GFP, Ad-SIK2 together with Ad-p300 or Ad-p300 S89A as indicated. Quantification of luminescent intensity of (**C**) was shown. (**F**) Immunoblot showing recovery of PPARα from IPs of p300 prepared from liver from (**C**). (**G**) Schematic of proposed mechanism in hepatocytes.

Given that SIK2 directly phosphorylates p300 at Ser89 to inhibit p300 activity^5^; we ask if SIK2 regulates PPARα activity through p300. Fasting induced dephosphorylation of p300 at Ser89 while feeding signal reversed it in liver (Fig. 4A), correlating with SIK2 activity^4^. Indeed, overexpression of SIK2 increased p300 phosphorylation at Ser89, while knockdown of SIK2 decreased it (Fig. 4B). Conversely, p300-PPARα interaction was enhanced under fasting condition while suppressed by feeding signal (Fig. 4A). Moreover, in liver from Ad-SIK2 expressed mice, p300-PPARα interaction was inhibited, while in Ad-SIK2i expressed liver, p300-PPARα interaction was enhanced (Fig. 4B). These data indicated that p300 phosphorylation by SIK2 regulated p300 binding to PPARα. Supporting this notion, SIK2-phosphorylation-defect mutant p300 (p300 S89A) interacted with PPARα with higher affinity comparing to wild type p300 (Fig. 4F). When expressed in HepG2 cells, p300 S89A promoted WY14643 plus FSK-induced *Fgf21*-PPRE-luc activity more dramatically comparing to WT p300 (Fig. S3A).

To further evaluate the role of p300 phosphorylation by SIK2 on PPARα activity, SIK2 was co-expressed with either WT p300 or p300 S89A in HepG2 cells. WY14643 plus FSK-induced *Fgf21*-PPRE-luc activity was enhanced by both WT and p300 S89A as expected (Fig. S3A). However, co-expression of SIK2 repressed WT p300-mediated PPARα activation but not p300 S89A-mediated PPARα activation (Fig. S3A). Conversely, knockdown of SIK2 in HepG2 enhanced WY14643 plus FSK-induced *Fgf21*-PPRE-luc activity; however, co-inhibition of p300 expression with SIK2 counteracted the stimulatory activity of SIK2 knockdown on *Fgf21*-PPRE-luc activity (Fig. S3B). Furthermore, when co-expressed in mouse liver, SIK2 was only able to inhibit hepatic *Aox*-PPRE-luc activity (Fig. 4C) as well as circulating ketone concentration (Fig. 4D) and PPARα target gene expression (Fig. 4E) when wild type p300 was present but not with p300 S89A mutant. Consistently, WT p300-PPARα interaction but not p300 S89A-PPARα interaction was inhibited by SIK2 expression (Fig. 4F). These results demonstrated that the phosphorylation at Ser89 and subsequent inhibition of p300 activity mediates the inhibition of PPARα activity by SIK2.

## Discussion

Gluconeogenesis and fatty acid oxidation/ketogenesis are the two major pathways induced by fasting glucagon signal in liver, which contribute to glucose and lipid homeostasis, respectively. SIK2 plays an important role in regulating hepatic gluconeogenesis pathway as CRTCs and Class II HDACs, two major regulators of gluconeogenesis, are both phosphorylated and inhibited by SIKs^5^,^6^,^7^. In the present study, we extend the importance of SIK2 in modulating fasting metabolism by showing that ketogenesis is also regulated by SIK2. SIK2 phosphorylates p300 at Ser89 and suppresses its interaction with and activation of PPARα (Fig. 4G). Based on these results, modulation of p300 activity by SIK2 could serve as an attractive approach to treat obesity and type 2 diabetes-associated liver dyslipidemia.

LXXLL motif has been reported to be important for proteins to interact with transcription factors such as steroid and retinoid receptors^20^, PPARγ^21^ and FOXO1^22^. Here, we show that p300 also interacts with PPARα through conserved LXXLL motif and p300 Ser89 phosphorylation greatly inhibits p300-PPARα interaction in our study. The mechanism of this inhibition still remains unclear. One hypothesis could be that because p300 Ser89 is located quite near the LXXLL motif (Leu81-Leu85)^19^, Ser89 phosphorylation may allosterically inhibit LXXLL binding to PPARα, though this claim will require further investigation.

It has been reported that SIK2 inhibited p300 activity could modulate multiple functions in liver. p300 promotes CRTC2 acetylation and gluconeogenesis during fasting state. SIK2 inhibits gluconeogenesis through phosphorylation and inhibition of p300 activity^4^. p300 also promotes Carbohydrate-responsive element–binding protein (ChREBP) acetylation and lipogenesis during feeding. SIK2 phosphorylation abolishes p300 effect on lipogenesis and helps to prevent hepatic steatosis^23^. Our data provide a new target of SIK2-p300 regulation in ketogenesis. However, the specificity of p300 Ser89 phosphorylation by SIK2 on p300 substrates remains unclear and should be carefully investigated in future studies. SIK2 phosphorylation of p300 may have a broad impact on multiple signal pathways and organ functions under specific physiological conditions.

## Methods

### Cells, antibodies, and reagents

HepG2 cells were transfected with lipofectamine3000 from Thermo Fisher Scientific. Anti-PPARα antibody was purchased from Santa Cruz (1:500 dilution). Anti-p300 (1:1000 dilution) and Anti-α-Tubulin antibodies were purchased from Abcam (1:5000 dilution). Anti-SIK2 (1:500 dilution) and Anti-pSer89 p300 (1:500 dilution) antibodies were purchased from Sigma. WY14643 and FSK were purchased from Sigma.

### Animals and adenovirus

8–10-week-old male C57BL/6J mice were purchased from Shanghai Laboratory Animal Center, CAS and were adapted to colony cages with 12 h light/dark cycle in a temperature-controlled environment for 1 week before study. 1 × 10^9^ plaque forming units (pfu) Ad-*Aox*-luc; 5 × 10^7^ pfu Ad-RSV-β-gal (Rous sarcoma virus promoter); 3 × 10^8^ pfu Ad-GFP, Ad-SIK2, Ad-p300, Ad-p300 S89A, Ad-p300 LXXAA; 1 × 10^9^ pfu Ad-unspecific RNAi (USi), Ad-SIK2 RNAi (SIK2i), Ad-p300 RNAi (p300i) were delivered by tail-vein injection. For *in vivo* imaging, mice were fasted for 48 h and refed for 2 h and imaged on day 3–5 after adenovirus delivery. Before imaging, mice were injected intraperitoneally with 50 mg/kg Nembutal (Abbott Laboratories) and 100 mg/kg sterile firefly D-luciferin (Biosynth AG). Mice were imaged on the IVIS 100 Imaging System, and analyzed with Living Image software (Xenogen) as described^5^. All animal studies were approved by the Animal Experiment Committee of Tongji University and in accordance with the guidelines of school of medicine, Tongji University.

### *In vitro* analysis

Mouse tissues were sonicated at 4 °C, centrifuged and supernatants were reserved for β-gal activity, protein determinations, SDS–PAGE analysis and quantitative PCR analysis. Protein expression or knockdown levels in mouse liver were shown in Fig. S4. Levels of serum total ketone body were determined using commercially available kits from WAKO.

### Luciferase reporter assay

HepG2 cells were transfected with *Fgf21*-PPRE-Luc reporter, RSV-β-gal, and indicated plasmids for 48 h and luciferase assays were performed as described^24^.

### RT-PCR and immunoblot

Total RNA was isolated by using TRIzol reagent and reverse transcription was done using FastQuant RT kit from Tiangen. Real-time PCR was carried out with SuperReal SYBR Green from Tiangen and Lightcycler 96 from Roche. Immunoblot and immunoprecipitation were performed as described^24^. All gels were run under the same experimental conditions.

### Statistical analysis

All studies were performed on at least three independent occasions. Results were reported as mean ± s.e.m. The comparison of two different groups was carried out using two-tailed unpaired Student’s t-test. One-way ANOVA was used to compare more than two groups. Differences were considered statistically significant at *p < 0.05 and **p < 0.01.

## Additional Information

**How to cite this article**: Zhang, Z.-N. *et al*. SIK2 regulates fasting-induced PPARa activity and ketogenesis through p300. *Sci. Rep*. **6**, 23317; doi: 10.1038/srep23317 (2016).

## Supplementary Material

Supplementary Information
