# Supplementary material for: SIK2 regulates fasting-induced PPARα activity and ketogenesis through p300
Source: Sci Rep. 2016 Mar 17;6:23317. doi: 10.1038/srep23317 (PMC4794759; doi:10.1038/srep23317)

SIK2 regulates fasting-induced PPARα activity and ketogenesis through p300

Zhen-Ning Zhang, Lulu Gong, Sihan Lv, Jian Li, Xiaolu Tai, Wenqi Cao, Bing Peng, Shen Qu, Weida Li, Chao Zhang, Bing Luan

Supplemental Figure Legend

Figure S1. SIKs inhibit PPARα activation in HepG2 cells.

A, Relative expression of SIKs family members (SIK1, SIK2 and SIK3) mRNA in liver measured by Q-PCR. B, *Fgf21*-PPRE-luciferase reporter activity in HepG2 cells co-transfected with PPARα and GFP, SIK1, SIK2, or SIK3 as indicated. Effect of FSK plus WY14643 was shown. C, Immunoblot showing SIK1, SIK2 and SIK3 expression in (B). D, *Fgf21*-PPRE-luciferase reporter activity in HepG2 cells co-transfected with PPARα and GFP, SIK2, dominant positive SIK2 (SIK2 S587A), or kinase inactive SIK2 (SIK2 K49M) as indicated. Effect of FSK plus WY14643 was shown. E, Immunoblot showing SIK2, SIK2 S587A and SIK2 K49M expression in (D). F, *Fgf21*-PPRE-luciferase reporter activity in HepG2 cells co-transfected with PPARα and USi or SIK2i as indicated. Effect of FSK plus WY14643 was shown. G, Immunoblot showing endogenous SIK2 expression in (F). H-I, Effect of WY14643 plus FSK on mRNA amounts of ketogenic genes expression from HepG2 cells transfected with GFP, SIK2 (D) or USi, SIK2i (E).

Figure S2. P300 promotes PPARα activation in HepG2 cells.

A, Effect of WY14643 plus FSK on mRNA amounts of ketogenic genes expression

from HepG2 cells transfected with GFP, p300, p300 S89A or p300 LXXAA as indicated. B, *Fgf21*-PPRE-luciferase reporter activity in HepG2 cells co-transfected with PPARα and GFP, p300, p300 S89A or p300 LXXAA as indicated. Effect of FSK plus WY14643 was shown. C, Immunoblot showing p300, p300 S89A or p300 LXXAA expression in (A). D, Effect of WY14643 plus FSK on mRNA amounts of ketogenic genes expression from HepG2 cells transfected with USi or p300i as indicated. E, *Fgf21*-PPRE-luciferase reporter activity in HepG2 cells co-transfected with USi or p300i as indicated. Effect of FSK plus WY14643 was shown. F, Immunoblot showing endogenous p300 expression in (D).

Figure S3. SIK2 regulates PPARα activation through p300 in HepG2 cells.

A, *Fgf21*-PPRE-luciferase reporter activity in HepG2 cells co-transfected with PPARα and expression vectors as indicated. Effect of FSK plus WY14643 was shown. B, *Fgf21*-PPRE-luciferase reporter activity in HepG2 cells co-transfected with PPARα and RNAi vectors as indicated. Effect of FSK plus WY14643 was shown.

Figure S4. Expression levels of adenovirus mediated protein expression or knockdown in liver.

A and B, Immunoblot showing SIK2 expression in liver of Ad-GFP, Ad-SIK2 (HA-tagged) (A) or Ad-USi, Ad-SIK2i (B) infected mice. C and D, Immunoblot showing p300 expression in liver of Ad-GFP, Ad-p300 (HA-tagged), Ad-p300 LXXAA (HA-tagged) (C) or Ad-USi, Ad-p300i (D) infected mice. E, Immunoblot

showing SIK2 and p300 expression in mouse liver infected with indicated adenovirus.

SIK2 regulates fasting-induced PPARα activity and ketogenesis through p300

Zhen-Ning Zhang, Lulu Gong, Sihan Lv, Jian Li, Xiaolu Tai, Wenqi Cao, Bing Peng, Shen Qu, Weida Li, Chao Zhang, Bing Luan

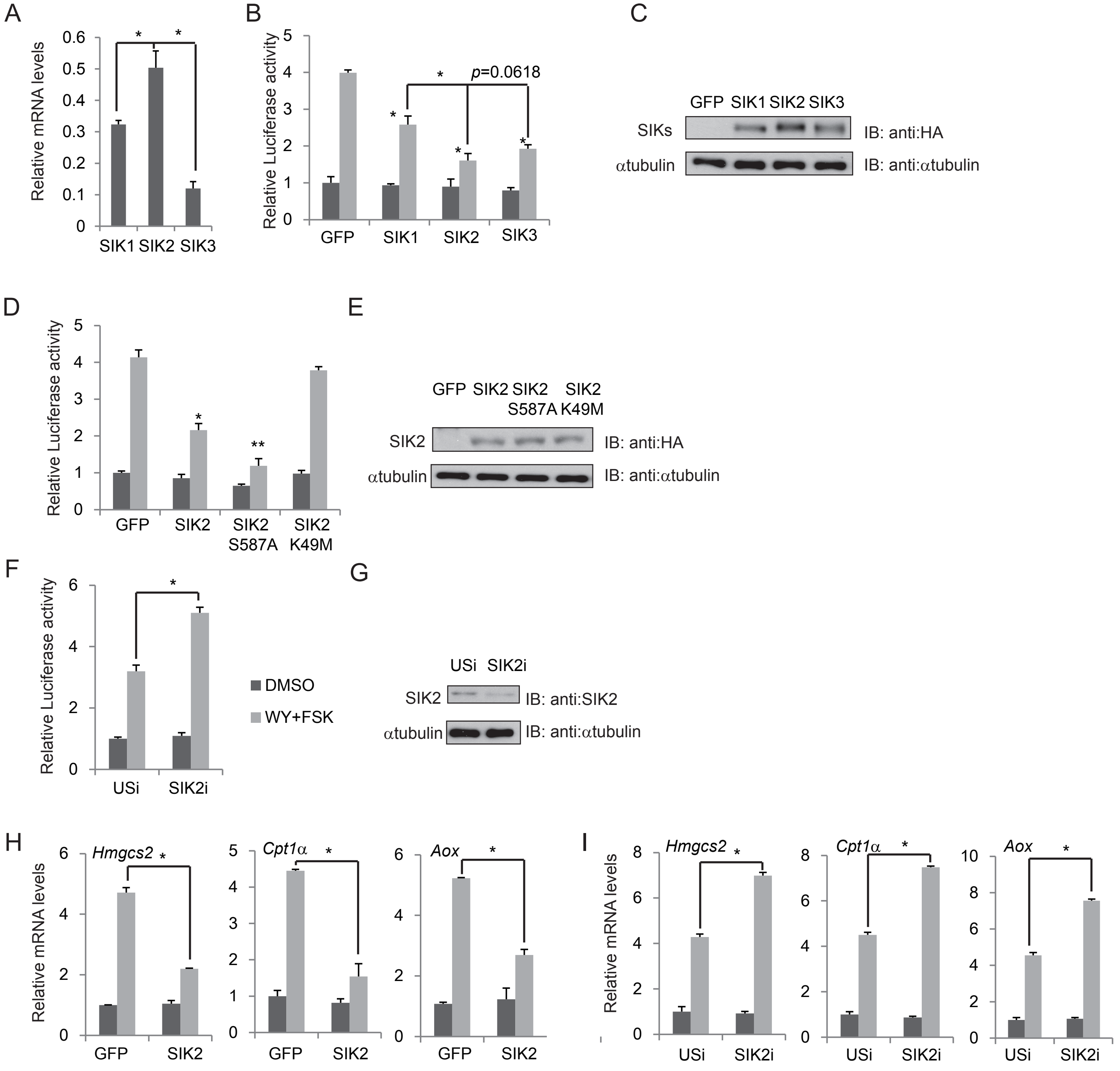

SIK2 regulates fasting-induced PPARα activity and ketogenesis through p300

Zhen-Ning Zhang, Lulu Gong, Sihan Lv, Jian Li, Xiaolu Tai, Wenqi Cao, Bing Peng, Shen Qu, Weida Li, Chao Zhang, Bing Luan

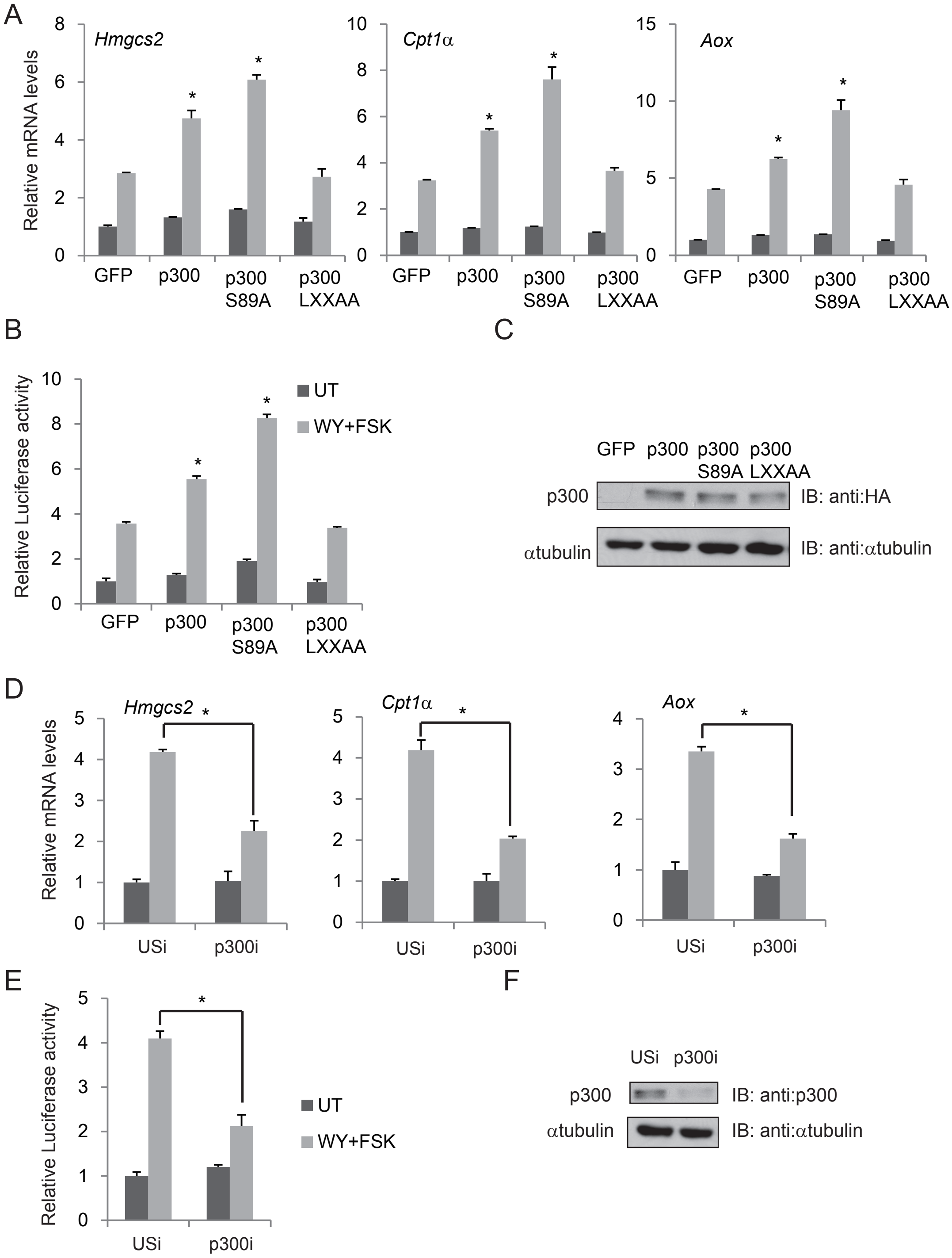

SIK2 regulates fasting-induced PPARα activity and ketogenesis through p300

Zhen-Ning Zhang, Lulu Gong, Sihan Lv, Jian Li, Xiaolu Tai, Wenqi Cao, Bing Peng,

Shen Qu, Weida Li, Chao Zhang, Bing Luan

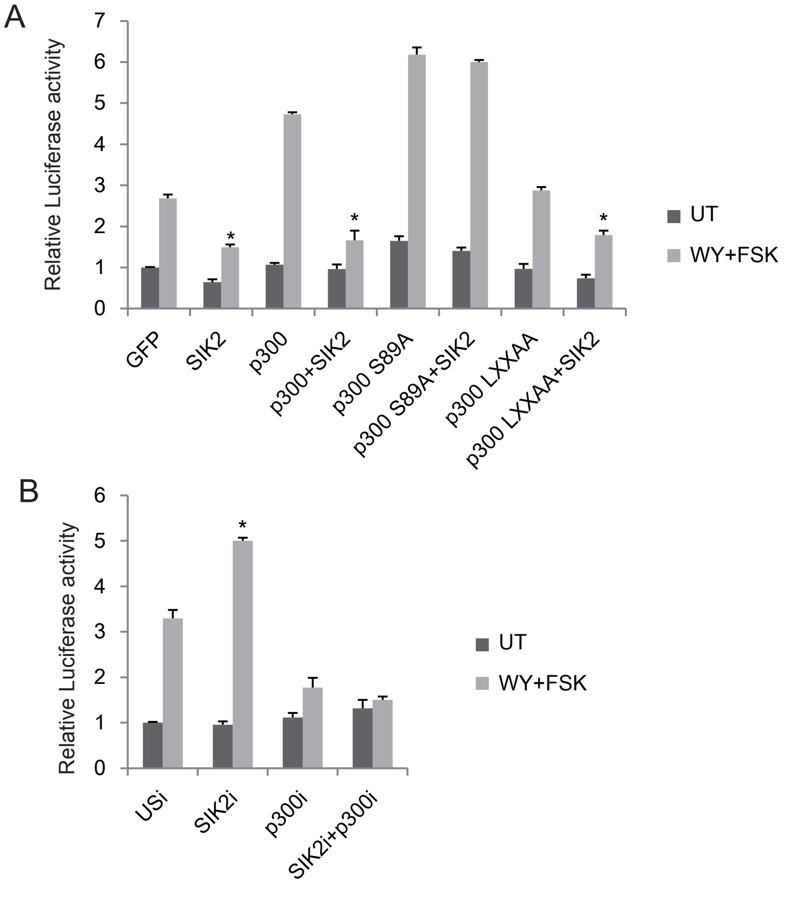

SIK2 regulates fasting-induced PPARα activity and ketogenesis through p300

Zhen-Ning Zhang, Lulu Gong, Sihan Lv, Jian Li, Xiaolu Tai, Wenqi Cao, Bing Peng, Shen Qu, Weida Li, Chao Zhang, Bing Luan

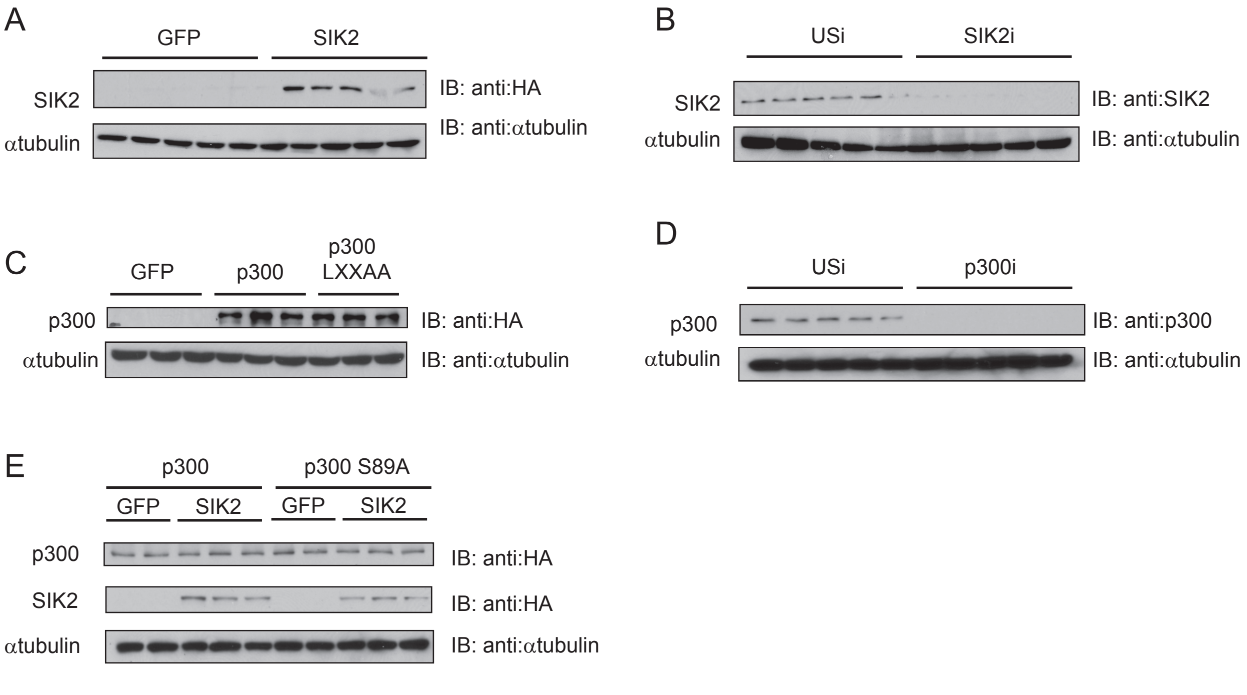

Supplement: Supplementary Information [file srep23317-s1.pdf]
